# Guidelines Disconcordance in Acute Bipolar Depression: Data from the National Bipolar Mania Pathway Survey (BIPAS) in Mainland China

**DOI:** 10.1371/journal.pone.0096096

**Published:** 2014-04-24

**Authors:** Zuowei Wang, Keming Gao, Wu Hong, Mengjuan Xing, Zhiguo Wu, Jun Chen, Chen Zhang, Chengmei Yuan, Jia Huang, Daihui Peng, Yong Wang, Weihong Lu, Zhenghui Yi, Xin Yu, Jingping Zhao, Yiru Fang

**Affiliations:** 1 Division of Mood Disorders, Department of Psychiatry, Shanghai Mental Health Center, Shanghai Jiao Tong University School of Medicine, Shanghai, China; 2 Division of Mood Disorders, Hongkou District Mental Health Center of Shanghai, Shanghai, China; 3 Mood and Anxiety Clinic in the Mood Disorders Program, Department of Psychiatry, University Hospitals Case Medical Center/Case Western Reserve University School of Medicine, Cleveland, Ohio, United States of America; 4 Institute of Mental Health, Peking University, Beijing, China; 5 Mental Health Institute, The Second Xiangya Hospital of Central South University, Changsha, Hunan, China; College of Mechatronics and Automation, National University of Defense Technology, China

## Abstract

With the recent attention to the importance of evidence-based medicine in psychiatry, a number of treatment guidelines have been published. This survey investigated prescribing pattern and predictors for guideline disconcordance in the acute treatment of bipolar depression across mainland China. Pharmacological treatments of 1078 patients with bipolar depression were examined. Guidelines disconcordance was determined by comparing the medication(s) patients were prescribed with the recommendation(s) in the guidelines of the Canadian Network for Mood and Anxiety Treatments. Predictors for guidelines discordance were analyzed with logistic regression. Of the 1078 patients, 50.2% patients were treated against treatment guidelines recommendations. The patients who were treated in general hospitals (OR = 1.53, 95% CI 1.18–1.97), with a depressive episode (OR = 1.67, 95% CI 1.27–2.19) and an older age at first onset (OR = 1.62, 95% CI 1.15–2.28) were more likely to receive guideline-disconcordant treatment than their counterparts. In contrast, the patients with current mental comorbidity, an older age at study entry, a longer duration of disease, and more frequent episodes in past year were less likely to receive guideline-disconcordant treatments than their counterparts with an OR of 0.43 (95% CI 0.24–0.77), 0.52 (95CI% 0.36–0.75), 0.48 (95% CI 0.36–0.65), and 0.50 (95% CI 0.38–0.64), respectively. Our finding suggested the disconcordance with treatment guidelines in patients with an acute bipolar depression is common under naturalistic conditions in mainland China, and the predicting factors correlated with guidelines disconcordance include both psychiatrist-specific (clinicians from general hospitals) and patient-specific features (a depressive episode at first onset, no current co-morbidity with mental disorders, a younger age at study entry, an older age at first onset, shorter duration of disease, and non-frequent episodes in past year).

## Introduction

There is growing recognition that bipolar disorder (BPD) is common, with bipolar disorder type I (BP-I) and type II (BP-II) affecting about 2% of the world’s population and subthreshold forms of the disorder affecting another 2% [1]–[2]. BPD is responsible for the loss of disability-adjusted life years (DALYs) more than all forms of cancer or major neurological conditions, primarily because of its early onset and chronicity across the life span [2]. Bipolar disorder is also a leading cause of premature mortality due to suicide and comorbid medical conditions such as diabetes and cardiovascular diseases [3]–[4]. Moreover, the recurrent nature of manic and depressive episodes often leads to high direct and indirect health care costs [5]–[6].

With recent attention to the importance of evidence-based medicine in psychiatry, a number of treatment guidelines have emerged to aid clinicians to make clinical decisions. Several practice guidelines have been published for the treatment of patients with BPD, which include guideline of the American Psychiatric Association [7], the British Psychological Society [8], the Chinese Medical Association [9], the World Federation of Societies of Biological Psychiatry [10]–[12], the Health Ministry of Singapore [13], and the Canadian Network for Mood and Anxiety Treatments (CANMAT) and the International Society for Bipolar Disorders (ISBD) [14]. Meanwhile, there is evidence that greater provider adherence to treatment guideline recommendations was associated with a greater reduction in symptoms and greater improvement in the outcome of diseases [15]–[16].

However, recent advances in pharmacological treatment for patients with bipolar disorder remained quite modest, and the treatment of bipolar depression was still a major challenge [17]. In addition, during routine clinical practice, the concordance with evidence-based guidelines in the treatment of BPD was typically low; and personal experience appeared in the foreground for the choice of therapies [16], [18]–. Until now, there has never been a study on the guideline-concordance for the treatment of BPD in China. This report is to present the data of a national survey (Bipolar Mania Pathway Survey, BIPAS) on the acute treatment of bipolar depression in routine clinical practice in Mainland China.

## Patients and Methods

### Patient Population

The BIPAS study was conducted in 26 sites (15 psychiatric hospitals and 11 psychiatric departments of general hospitals) across mainland China from the November of 2012 to the January of 2013. All procedures were reviewed and approved by the institutional review boards of Shanghai Mental Health Center. Written informed consents were obtained from each participant before any study-related procedure was performed. For the participants who had a compromised capacity/ability to consent, the written informed consents were obtained from the legally authorized representatives. Diagnosis of bipolar disorder was ascertained with *ICD-10* (International Classification of Disease –10 Edition) diagnostic criteria for bipolar affective disorder. Both inpatients and outpatients who presented with an acute mood episode or in remission were consecutively screened for this study.

### Clinical Assessments

All patients who were enrolled in the study received a single-round survey. During the screening visit, demographic characteristics, psychiatric and medical histories, and other variables of interest, were obtained from patients, their family members, and medical records by research psychiatrists and research assistants. The prescribing information (name of psychotropic agents) for a recent depressive episode was retrospectively obtained from patients’ medical records.

### Disconcordance Determination

Recommendations for pharmacological treatment of acute bipolar depression by CANMAT guidelines [14] were used to determine the treatment(s) as guideline concordance or disconcordance. The use of these guidelines as “standard” was justified because there was no update on treatment-guidelines for bipolar disorder in China since 2007 and the guidelines of CANMAT and ISBD for bipolar disorders were commonly recommended during continuing medical education and clinical practice in Mainland China. Lithium, lamotrigine, divalproex, quetiapine, or olanzapine monotherapy as well as the combination treatment of these agents with other psychotropic agent(s) were considered to be guideline-concordant in this survey. Therefore, other treatment option(s) was considered as guidelines disconcordance.

### Data Analysis

Demographics characteristics, clinical features and treatment options for a recent depressive episode were analyzed with descriptive statistics. The logistic regression was performed to study the predictors of guideline disconcordance. The variables analyzed in the regression model included gender (male vs. female), hospital category (general vs. psychiatric), mood state at first onset (hypomanic/manic episode vs. depressive episode), current co-morbidity with mental disorders (no vs. yes), current co-morbidity with physical disorders (no vs. yes), family history of mental disorders (no vs. yes), age at study entry (≤30 years vs. >30 years), age at first episode (≤23 years vs. >23 years), duration of disease (≤4 years vs. >4 years), and the number of episodes in past year (≤1 time vs. >1 time). The same variables were also analyzed for the predictors of antidepressant monotherapy. Those continuous variables were converted to categorical variables based on their medians. Statistical significance was set at α = 0.05, two-tailed, in order to detect potentially clinically meaningful associations. Given the exploratory nature of the study, no adjustment for multiple comparisons was made.

## Results

### Demographics and Clinical Characteristics

The majority of 1078 patients were Han Chinese and with depression as the first episode of the onset of bipolar disorder (Table 1). Gender and sources of patients were similar. Most patients did not have current mental or physical comorbidity, or family history of mental disorders. The median age at the study entry was 30.5 years old. The median age at the onset of first mood episode was 23 years old. The median duration of disease was 4 years. The median number of mood episodes in past year was one episode (Table 1).

**Table 1:** Demographics and clinical characteristics of patients with acute bipolar depression.

Characteristic	N	%
Gender		
-Male	559	51.9
-Female	519	48.1
Race		
-Han	1059	98.2
-Other	19	1.8
Hospital category		
-General	508	47.1
-Psychiatric	570	52.9
Mood state at first onset		
-Hypomanic/manic episode	344	31.9
-Depressive episode	734	68.1
Current co-morbidity		
-Mental disorders	59	5.5
-Physical disorders	95	8.8
Family history of mental disorders	902	83.7
	Median	IQR
Age at study entry (years)	30.5	22.0–42.0
Age at first-onset episode (years)	23.0	18.0–32.0
Duration of disease (years)	4.0	1.0–9.0
Number of episodes in past year	1.0	1.0–2.0

### Patterns of Pharmacological Treatment for Acute Bipolar Depression

As shown in Table 2, of the 1078 patients, 452 (41.9%) patients received treatment with one psychotropic agent; and antidepressant monotherapy was the most commonly prescribed treatment (n = 341, 31.6%). The second most commonly prescribed treatment was the combination of two psychotropic agents (n = 330, 30.6%). Two drug therapy included a mood stabilizer plus an antidepressant (140 patients, 13.0%), a mood stabilizer plus an atypical antipsychotic (89 patients, 8.3%), olanzapine or quetiapine plus an antidepressant (63 patients, 5.8%), and one other atypical antipsychotic (AAP) plus an antidepressant (34 patients, 3.2%). One hundred and sixty-three (15.1%) patients received the combination of three drugs with a mood stabilizer plus an atypical antipsychotic plus an antidepressant as the most common combination in this strategy. Meanwhile, 133 (12.3%) patients did not receive any psychotropic agent (Table 2). Totally, 541 (50.2%) patients’ treatments did not follow the treatment guidelines (no agent n = 133, an antidepressant n = 341, an AAP n = 33, and an AAP plus an antidepressant n = 34).

**Table 2:** Patterns of pharmacological treatments in patients with acute bipolar depression.

Treatment Strategies	N	%
*No agent*	*133*	*12.3*
*One agent*	*452*	*41.9*
MS^a^	52	4.8
Olanzapine/Quetiapine	26	2.4
Antidepressant	341	31.6
AAP^b^	33	3.1
*Two agents*	*330*	*30.6*
MS+Olanzapine/Quetiapine/AAP	89	8.3
MS+Antidepressant	140	13.0
Olanzapine/Quetiapine+Antidepressant	63	5.8
AAP+Antidepressant	34	3.2
Olanzapine+Quetiapine or Olanzapine/Quetiapine+AAP	4	0.4
*Three agents*	*163*	*15.1*
MS+Olanzapine/Quetiapine/AAP+Antidepressant	145	13.5
Olanzapine/Quetiapine+APP+Antidepressant	6	0.6
MS+Olanzapine/Quetiapine+APP	12	1.1

a MS, mood stabilizers (including lithium, divalproex and lamotrigine).

b AAP, atypical antipsychotics (excluding Olanzapine/Quetiapine).

### Predictors of Guideline-Disconcordant Treatments in Acute Bipolar Depression

Among the 10 variables considered for logistic regression analysis, 7 of them including hospital category, mood state at first onset, current co-morbidity with mental disorders, age at study entry, age at first onset, duration of disease, and number of episodes in past year remained in the model after a stepwise model building process. The patients who were treated in general hospitals were more likely to receive guideline-disconcordant treatment than patients treated in psychiatric hospitals with an OR of 1.53 (95% CI 1.18–1.97). Similarly, patients with a depressive episode and an older age (>23 years) at the onset of first mood episode were also more likely to receive guideline-disconcordant treatment than their counterparts with an OR of 1.67 (95% CI 1.27–2.19) and 1.62 (95% CI 1.15–2.28), respectively. In contrast, patients with current mental comorbidity, an older age (>30 years) at study entry, longer duration of disease (>4 years), and more frequent episodes (more than one time) in past year were less likely to receive guideline-disconcordant treatments than their counterparts with an OR of 0.43 (95% CI 0.24–0.77), 0.52 (95CI% 0.36–0.75), 0.48 (95% CI 0.36–0.65), and 0.50 (95% CI 0.38–0.64), respectively (Table 3).

**Table 3:** Predictors for guideline-disconcordant treatments and antidepressant monotherapy in acute bipolar depression.

Predictors	Wald *χ^2^*	*P-value*	OR	95% CI
	*Guideline-disconcordant treatments*			
General hospitals	10.58	0.001	1.53	1.18–1.97
Depressive episode at first onset	13.40	0.000	1.67	1.27–2.19
Current co-morbidity with mental disorders	7.93	0.005	0.43	0.24–0.77
Age at study entry (>30 years)	12.03	0.001	0.52	0.36–0.75
Age at first onset (>23 years)	7.51	0.006	1.62	1.15–2.28
Duration of disease (>4 years)	24.11	0.000	0.48	0.36–0.65
Number of episodes in past year (>1 time)	27.48	0.000	0.50	0.38–0.64
	*Antidepressant monotherapy*			
Depressive episode at first onset	21.69	0.000	2.06	1.52–2.79
Family history with mental disorders	8.23	0.004	0.60	0.43–0.85
Age at study entry (>30 years)	11.04	0.001	0.51	0.35–0.76
Age at first onset (>23 years)	19.11	0.000	2.24	1.56–3.22
Duration of disease (>4 years)	3.60	0.058	0.74	0.55–1.01
Number of episodes in past year (>1 time)	11.33	0.001	0.62	0.47–0.82

### Predictors of Antidepressant Monotherapy in Acute Bipolar Depression

Among the 10 variables considered for logistic regression analysis, 6 of them including mood state at first onset, family history with mental disorders, age at study entry, age at first onset, duration of disease, and number of episodes in past year, remained in the model after a stepwise model building process. A depressive episode at first onset and an older age (>23 years) at the onset of first mood episode significantly increased the risk for antidepressant monotherapy with an OR of 2.06 (95% CI 1.52–2.79) and an OR of 2.24 (95% CI 1.56–3.22), respectively. In contrast, a family history with mental disorder, an older age (>30 years) at study entry, and more frequent episodes (more than one time) in past year, were associated with a significant decrease in the risk for antidepressant monotherapy with an OR of 0.60 (95% CI 0.43–0.85), 0.51 (95% CI 0.35–0.76) and 0.62 (95% CI 0.47–0.82), respectively. A longer duration (>4 years) of disease was associated with a trended decrease in the risk for antidepressant monotherapy with an OR of 0.74 (95% CI 0.55–1.01) (Table 3).

## Discussion

To our knowledge, this is the first national survey to investigate the current practice of pharmacological treatments and guideline concordance for acute bipolar depression in Mainland China. The inclusion of both inpatients and outpatients from both psychiatric hospitals and general hospitals increase the generalizability of this study. According to CANMAT guideline recommendations without considering sequenced treatment alternatives, the disconcordant rate with guidelines in the present study was more than 50% (Table 2).

The major pattern of guideline-disconcordant treatments in this cross-section survey was antidepressant monotherapy (31.6%). Other minor patterns included not prescribing any psychotropic agent (12.3%), atypical antipsychotic (excluding olanzapine and quetiapine) monotherapy, and the combination treatment of atypical antipsychotic plus antidepressant (6.3%) with the exception of olanzapine-fluoxetine combination. The reported concordant rate of treatment guidelines for bipolar disorders worldwide were from 17.0% to 87.5% among different populations, which depends on types of disease and episode, severity of episode, comorbid psychotic features, duration of treatment and intervention for adherence to guidelines [23]. In the Systematic Treatment Enhancement Program for Bipolar Disorder (STEP-BD) study from United States, the rates of guidelines concordance were 81.8% for mixed episodes, 81.9% for hypomanic/manic episodes, and 83.4% for depressive episodes, respectively [18]. Thus the disconcordant rate to treatment guidelines in our survey was modest compared with those previously reported in other countries. Since the CANMAT guidelines were introduced from western countries, a possible factor may be derived from the differences between Chinese and western cultures.

The probable factors predicting guidelines disconcordance are both psychiatrist-specific and patient-specific. The practice type and experience of clinicians were confirmed to be important factors affecting clinical decision and guideline concordance [15], [22]. Those practitioners who did not comply with guidelines believed that those guidelines did not address particular features of their clinical patient populations [19]. In Mainland China, non-psychiatric specialists (such as neurologists) in general hospitals usually prescribe medication(s) for patients with mood disorders, and these physicians receive different continuing medicine education compared to psychiatrists, which leads them to prescribe psychotropic agents by personal experiences more than psychiatrists. In addition, those non-psychiatric specialists prescribing psychotropic agents in general hospitals are usually older with longer duration of practice, which refer less to guidelines as compared with younger physicians [22].

The finding of a depressive episode at first onset associated with increased risk for guidelines-disconcordance is consistent with previous studies [24]. One possible reason is that misdiagnosis of bipolar depression as unipolar depression with guideline-disconcordant treatments [25]. Since our study focusing on the recent depressive episode, the previous diagnose(s) could not be verified. Therefore, the proportion of discorntant-treatments due to misdiagnosis or correct diagnosis with inappropriate treatments is difficult to be determined.

And patients with late onset of depression were characterized by a higher prevalence of stressful life events preceding onset compared to patients with early onset [26], so some patients with an older age of first mood episode would be treated with psychological therapy, not psychotropic agent in this survey. In addition, an earlier age of first onset (e.g. earlier than 25 years old) is one of characteristics distinguishing bipolar depression from first depressive episode and [24], [27]. Thus, an older age of first mood episode may also contribute to undiagnosed bipolar disorder with inappropriate treatments.

Among the 4 factors associated with the decreased risk for disconcordant treatments, at least 2 of them including a longer duration of bipolar illness, and more frequent episodes in the last 12 months were due to one possible reason providing more opportunities for clinician(s) to make a correct diagnosis. An older age at the study entry also provided more opportunities for diagnosis, but its independence from the duration of illness and other factors suggests that older age may affect diagnosis and treatments differently compared to those with younger age. Studies have shown that early onset bipolar disorder commonly has atypical presentation of mania/hypomania [24], [28]–[29]. One plausible explanation for the association between older age and decreased risk for disconcordant-treatments is that patients with older age at the study entry may have typical presentation of mania/hypomania and more insight about their illness so that they can provide more reliable information for a correct diagnosis.

The finding of the decreased risk for disconcordant-treatments in patients with psychiatric comorbidity is unexpected. Previous studies from the United States showed that bipolar patients with psychiatric comorbidities were commonly inappropriately treated [30]–[31]. This discrepancy could be due to the following. First, the prevalence of current psychiatric comorbidity in our study was only 5.5%, which is much lower than previous studies [32]–[33]. This low rate of comorbidity may reduce the risk for misdiagnosis [34]. Second, in China, psychiatric patients are commonly treated by psychiatric professionals, especially those with multiple psychiatric presentations. The comorbidity may alert clinicians to probe questions about mania/hypomania.

The results of the high frequent use of antidepressants, either as adjunctive therapy (67.6%) or monotherapy (31.6%), in the present study are consistent previous studies from different countries [23], [35]–[37]. There have been reports that the increased use of antidepressants in bipolar disorder was related to substantial depressive illness burden and suicidality [38], being women, and diagnosis of BP-II or not-otherwise-specified (NOS) [39]. The role of antidepressants in the acute treatment of bipolar depression remains controversial [14], [17]. More recently, ISBD Task Force Report on Antidepressant Use in Bipolar Disorders concluded that because of limited data, the task force could not make broad statements endorsing antidepressant use but acknowledged that individual bipolar patients may benefit from antidepressants. Regarding safety, serotonin reuptake inhibitors and bupropion has lower rates of manic switch than tricyclic and tetracyclic antidepressants and norepinephrine-serotonin reuptake inhibitors. The frequency and severity of antidepressant-associated mood elevations appear to be greater in BP-I than BP-II. Therefore, in BP-I patients antidepressants should be prescribed only as an adjunct to mood-stabilizing medications [40]. The combination therapy for acute bipolar depression is a common practice [7]. The most common combinations were at least two agents, including mood stabilizers, atypical antipsychotics and antidepressants [41]. The combination of two (30.6%) or more psychotropic agents (three agents 15.1%) for acute bipolar depression in the present study was consistent with previous studies [23], [37]. The most common prescribing patterns of mood stabilizer plus antidepressant (13.0%) and mood stabilizer plus antipsychotic and antidepressant (13.5%) were also consistent with previous studies [11], [14]. Because our study was cross-sectional and retrospective, the role of combination therapy during the course of depression was unknown. However, in a study examined the role of combination pharmacotherapy when monotherapy was unsuccessful, Post et al. found that complex medication regimens were required to achieve a sustained response for 6 months during naturalistic outpatient treatment of bipolar disorder [42].

### Limitations

Several limitations of this study should be considered. First, this study was carried out in 26 large-scale psychiatric and general hospitals across mainland China. They are all located in the provincial capital cities or municipalities and directly under the central governments’ control. The current practice for the treatment of patients with bipolar disorder in small and medium-sized cities, village or community was not included in this study. Second, this study was a cross-sectional and retrospective investigation based on medical records, which did not allow us to study the changes in the treatment regimes for patients with bipolar depression. Some psychotropic agents could be the continuation from previous treatment regimes for previous mood episodes. Therefore, some agents might not be used specially for the recent depressive episodes. Third, the studied demographics and clinical characteristics were most patient-specific factors, and inpatients and outpatients were not discriminated. Clinician-specific factors such as age, education level, and training experience were reported to affect prescribing pattern and guideline concordance [15], [19], [22], but in the present study, they were not available.

## Conclusions

The findings showed that the guideline-disconcordant treatments in patients with acute bipolar depression were very common (more than 50%) under naturalistic conditions, and antidepressant monotherapy was the major contribution to so high disconcordant rate in this surveyed population. The predicting factors correlated with guidelines disconcordance include both psychiatrist-specific (clinicians from general hospitals) and patient-specific features (a depressive episode at first onset, no current co-morbidity with mental disorders, a younger age at study entry, an older age at first onset, shorter duration of disease, and non-frequent episodes in past year). While those patient-specific features (except current mental comorbidity) are also predicting factors for antidepressant monotherapy. Considering the potentially hazardous effects of inappropriate pharmacotherapy in this population, continuing education and training to avoid the misdiagnosis of BPD and irrational psychotropic treatment, and to close the gap between treatment guidelines and clinical practice, are necessary in mainland China.

## References

[1] Merikangas KR, Akiskal HS, Angst J, Greenberg PE, Hirschfeld RM, et al (2007) Lifetime and 12-month prevalence of bipolar spectrum disorder in the National Comorbidity Survey replication. Arch Gen Psychiatry 64: 543–552. 17485606 10.1001/archpsyc.64.5.543 PMC1931566

[2] Merikangas KR, Jin R, He JP, Kessler RC, Lee S, et al (2011) Prevalence and correlates of bipolar spectrum disorder in the world mental health survey initiative. Arch Gen Psychiatry 68: 241–251. 21383262 10.1001/archgenpsychiatry.2011.12 PMC3486639

[3] Osby U, Brandt L, Correia N, Ekbom A, Sparén P (2001) Excess mortality in bipolar and unipolar disorder in Sweden. Arch Gen Psychiatry 58: 844–850. 11545667 10.1001/archpsyc.58.9.844

[4] Kupfer DJ (2005) The increasing medical burden in bipolar disorder. JAMA 293: 2528–2530. 15914754 10.1001/jama.293.20.2528

[5] Kleinman L, Lowin A, Flood E, Gandhi G, Edgell E, et al (2003) Costs of bipolar disorder. Pharmacoeconomics 21: 601–622. 12807364 10.2165/00019053-200321090-00001

[6] Dilsaver SC (2011) An estimate of the minimum economic burden of bipolar I and II disorders in the United States: 2009. J Affect Disord 129: 79–83. 20888048 10.1016/j.jad.2010.08.030

[7] American Psychiatric Association (2002) Practice guideline for the treatment of patients with bipolar disorder (revision). Am J Psychiatry 159 (Suppl. 4)1–50. 11958165

[8] National Collaborating Center for Mental Health (2006) Bipolar Disorder: The Management of Bipolar Disorder in Adults, Children and Adolescents, in Primary and Second Care. Leicester (UK): British Psychological Society. 21796830

[9] Shen QJ, Liu TB, Zhang HY, Ma X, Wang GH, et al.. (2007) Practice guideline for prevention and treatment of bipolar disorder (in Chinese). Beijing (China): Chinese Medical Association.

[10] Grunze H, Vieta E, Goodwin GM, Bowden C, Licht RW, et al (2009) The World Federation of Societies of Biological Psychiatry (WFSBP) guidelines for the biological treatment of bipolar disorders: update 2009 on the treatment of acute mania. World J Biol Psychiatry 10: 85–116. 19347775 10.1080/15622970902823202

[11] Grunze H, Vieta E, Goodwin GM, Bowden C, Licht RW, et al (2010) The World Federation of Societies of Biological Psychiatry (WFSBP) guidelines for the biological treatment of bipolar disorders: update 2010 on the treatment of acute bipolar depression. World J Biol Psychiatry 11: 81–109. 19347775 10.1080/15622970902823202

[12] Grunze H, Vieta E, Goodwin GM, Bowden C, Licht RW, et al (2013) The World Federation of Societies of Biological Psychiatry (WFSBP) guidelines for the biological treatment of bipolar disorders: update 2012 on the long-term treatment of bipolar disorder. World J Biol Psychiatry 14: 154–219. 23480132 10.3109/15622975.2013.770551

[13] Mok YM, Chan HN, Chee KS, Chua TE, Lim BL, et al (2011) Ministry of Health clinical practice guidelines: bipolar disorder. Singapore Med J 52: 914–918. 22159936

[14] Yatham LN, Kennedy SH, Parikh SV, Schaffer A, Beaulieu S, et al (2013) Canadian Network for Mood and Anxiety Treatments (CANMAT) and International Society for Bipolar Disorders (ISBD) collaborative update of CANMAT guidelines for the management of patients with bipolar disorder: update 2013. Bipolar Disord15: 1–44. 10.1111/bdi.12025 23237061

[15] Dennehy EB, Suppes T, Rush AJ, Miller AL, Trivedi MH, et al (2005) Does provider adherence to a treatment guideline change clinical outcomes for patients with bipolar disorder? Results from the Texas Medication Algorithm Project. Psychol Med 35: 1695–1706. 16194283 10.1017/S0033291705005933

[16] Bauer MS, Biswas K, Kilbourne AM (2009) Enhancing multiyear guideline concordance for bipolar disorder through collaborative care. Am J Psychiatry 166: 1244–1250. 19797436 10.1176/appi.ajp.2009.09030342

[17] Geddes JR, Miklowitz DJ (2013) Treatment of bipolar disorder. Lancet 381: 1672–1682. 23663953 10.1016/S0140-6736(13)60857-0 PMC3876031

[18] Dennehy EB, Bauer MS, Perlis RH, Kogan JN, Sachs GS (2007) Concordance with treatment guidelines for bipolar disorder: data from the systematic treatment enhancement program for bipolar disorder. Psychopharmacol Bull 40: 72–84. 18007570

[19] Perlis RH (2007) Use of treatment guidelines in clinical decision making in bipolar disorder: a pilot survey of clinicians. Curr Med Res Opin 23: 467–475. 17355728 10.1185/030079906X167444

[20] Smith TE, Levine SB, Hampel J (2008) A successful effort to improve adherence to treatment guidelines for bipolar disorder. Harv Rev Psychiatry 16: 210–213. 18569042 10.1080/10673220802160415

[21] Altınbaş K, Smith D, Oral ET (2011) Adherence to Turkish psychiatric association guideline for bipolar depression treatment in a specialized mood disordersoutpatient unit. Psychiatr Danub 23: 189–193. 21685859

[22] Samalin L, Guillaume S, Auclair C, Llorca PM (2011) Adherence to guidelines by French psychiatrists in their real world of clinical practice. J Nerv Ment Dis 199: 239–243. 21451347 10.1097/NMD.0b013e3182125d4c

[23] Paterniti S, Bisserbe JC (2013) Pharmacotherapy for bipolar disorder and concordance with treatment guidelines: survey of a general population sample referred to a tertiary care service. BMC Psychiatry 13: 211. 23941445 10.1186/1471-244X-13-211 PMC3751340

[24] Xiang YT, Zhang L, Wang G, Hu C, Ungvari GS, et al (2013) Sociodemographic and clinical features of bipolar disorder patients misdiagnosed with major depressive disorder in China. Bipolar Disord 15: 199–205. 23437963 10.1111/bdi.12052

[25] Xiang YT, Hu C, Wang G, Zheng QW, Fang YR, et al (2012) Prescribing patterns of antidepressants, antipsychotics and mood stabilizers in bipolar patients misdiagnosed with major depressive disorder in China. Hum Psychopharmacol 27: 626–631. 23027671 10.1002/hup.2262

[26] Bukh JD, Bock C, Vinberg M, Gether U, Kessing LV (2011) Differences between early and late onset adult depression. Clin Pract Epidemiol Ment Health 7: 140–147. 21866230 10.2174/1745017901107010140 PMC3158434

[27] Fang Y, Wang Z (2011) Current status and future trends in clinical research of bipolar disorder (in Chinese). Shanghai Archives of Psychiatry 23: 12–16.

[28] Azorin JM, Angst J, Gamma A, Bowden CL, Perugi G, et al (2012) Identifying features of bipolarity in patients with first-episode postpartum depression: findings from the international BRIDGE study. J Affect Disord 136: 710–715. 22044629 10.1016/j.jad.2011.10.003

[29] Masi G, Mucci M, Pfanner C, Berloffa S, Magazù A, et al (2012) Developmental pathways for different subtypes of early-onset bipolarity in youths. J Clin Psychiatry 73: 1335–1341. 23058936 10.4088/JCP.11m07504

[30] Simon NM, Otto MW, Weiss RD, Bauer MS, Miyahara S, et al (2004) Pharmacotherapy for bipolar disorder and comorbid conditions: baseline data from STEP-BD. J Clin Psychopharmacol 24: 512–520. 15349007 10.1097/01.jcp.0000138772.40515.70

[31] Gao K, Kemp DE, Conroy C, Ganocy SJ, Findling RL, et al (2010) Comorbid anxiety and substance use disorders associated with a lower use of mood stabilisers in patients with rapid cycling bipolar disorder: a descriptive analysis of the cross-sectional data of 566 patients. Int J Clin Pract 64: 336–344. 20456174 10.1111/j.1742-1241.2009.02284.x PMC3457056

[32] Simon NM, Otto MW, Wisniewski SR, Fossey M, Sagduyu K, et al (2004) Anxiety disorder comorbidity in bipolar disorder patients: data from the first 500 participants in the Systematic Treatment Enhancement Program for Bipolar Disorder (STEP-BD). Am J Psychiatry 161: 2222–2229. 15569893 10.1176/appi.ajp.161.12.2222

[33] Gao K, Wang Z, Chen J, Kemp DE, Chan PK, et al (2013) Should an assessment of Axis I comorbidity be included in the initial diagnostic assessment of mood disorders? Role of QIDS-16-SR total score in predicting number of Axis I comorbidity. J Affect Disord 148: 256–264. 23273550 10.1016/j.jad.2012.12.004

[34] Hirschfeld RM, Lewis L, Vornik LA (2003) Perceptions and impact of bipolar disorder: how far have we really come? Results of the national depressive and manic-depressive association 2000 survey of individuals with bipolar disorder. J Clin Psychiatry 64: 161–174. 12633125

[35] Baldessarini RJ, Leahy L, Arcona S, Gause D, Zhang W, et al (2007) Patterns of psychotropic drug prescription for U.S. patients with diagnoses of bipolar disorders. Psychiatr Serv 58: 85–91. 17215417 10.1176/ps.2007.58.1.85

[36] Carta MG, Aguglia E, Balestrieri M, Calabrese JR, Caraci F, et al (2012) The lifetime prevalence of bipolar disorders and the use of antidepressant drugs in bipolar depression in Italy. J Affect Disord 136: 775–780. 22030133 10.1016/j.jad.2011.09.041

[37] Greil W, Häberle A, Haueis P, Grohmann R, Russmann S (2012) Pharmacotherapeutic trends in 2231 psychiatric inpatients with bipolar depression from the International AMSP Project between 1994 and 2009. J Affect Disord 136: 534–542. 22134044 10.1016/j.jad.2011.10.033

[38] Goldberg JF, Brooks JO 3rd, Kurita K, Hoblyn JC, Ghaemi SN, et al (2009) Depressive illness burden associated with complex polypharmacy in patients with bipolar disorder: findings from the STEP-BD. J Clin Psychiatry 70: 155–162. 19210946 10.4088/jcp.08m04301 PMC10034852

[39] Lorenzo LS, Vázquez GH, Zaratiegui RM, Tondo L, Baldessarini RJ (2012) Characteristics of bipolar disorder patients given antidepressants. Hum Psychopharmacol 27: 486–491. 22927134 10.1002/hup.2253

[40] Pacchiarotti I, Bond DJ, Baldessarini RJ, Nolen WA, Grunze H, et al (2013) The International Society for Bipolar Disorders (ISBD) Task Force Report on Antidepressant Use in Bipolar Disorders. Am J Psychiatry 170: 1249–1262. 24030475 10.1176/appi.ajp.2013.13020185 PMC4091043

[41] Vieta E, Valentí M (2013) Pharmacological management of bipolar depression: acute treatment, maintenance, and prophylaxis. CNS Drugs 27: 515–529. 23749421 10.1007/s40263-013-0073-y

[42] Post RM, Altshuler LL, Frye MA, Suppes T, Keck PE Jr, et al (2010) Complexity of pharmacologic treatment required for sustained improvement in outpatients with bipolar disorder. J Clin Psychiatry 71: 1176–1186. 20923622 10.4088/JCP.08m04811yel

